# Unperceived bronchial bleeding complications during percutaneous dilatational tracheotomy: a case report and 3D simulation

**DOI:** 10.1186/s41205-025-00270-1

**Published:** 2025-05-30

**Authors:** Khalid Salem, Hendrik Drinhaus, Dominique Hart, Bernd W. Böttiger, Andrea U. Steinbicker, Bernhard Dorweiler, Fabian Dusse

**Affiliations:** 1 Department of Anesthesiology and Intensive Care Medicine, Faculty of Medicine and University Hospital Cologne, University of Cologne, Cologne, Germany; 2 Department of Vascular and Endovascular Surgery, Faculty of Medicine and University Hospital Cologne, University of Cologne, Cologne, Germany

**Keywords:** Guide wire, Bronchoscopy, Mucosal lesion, Thrombocytopenia

## Abstract

Percutaneous dilatational tracheostomy is an established technique for securing the airway in critically ill patients. One of the most common complications is bleeding around the incision or after injury to major vessels in anatomic proximity.

We report a case in which a thrombocytopenic patient experienced life-threatening bleeding during the procedure at the bifurcation between segmental bronchus 9 and 10, apparently caused by an unrecognized guide wire-induced mucosal lesion. Immediate extensive bronchoscopy and hemostatic interventions were required to ensure oxygenation. To better illustrate this complication, a patient-specific (1:1) three-dimensional model of the patient’s bronchial system was subsequently created using a 3D printer. In conclusion, 3d printing can help to visualize uncommon complications during intensive care interventions. It is recommended to advance the guide wire the guide wire only until the tracheal carina under bronchoscopic control.

Word count: 135.

## Introduction

The first reference of a tracheotomy can be found 1500 BC in the records of the Eber Papyrus [1]. The standardized surgical technique was described by Chevalier in 1909 [2]. Today, a common standard procedure is the percutaneous dilatational tracheostomy (PDT) according to Ciaglia, performed by two health care providers at bedside [3]. The PDT creates an opening in the anterior tracheal wall by dilation for a tracheostomy tube placement. This bedside procedure is particularly indicated for patients requiring long-term mechanical ventilation.

Sonography should be performed prior to the procedure to visualize the anatomy, in particular large blood vessels. In case large vessels are present, surgical tracheotomy is recommended.

The PDT procedure requires a Ciaglia Single Dilator Technique Procedural Kit. After sterile setup, the bronchoscope is inserted, and the endotracheal tube is first unblocked, pulled into the subglottic area, and blocked again at this level. A needle is then inserted into the trachea under bronchoscopic control between the second and third tracheal ring. After that, a guide wire is inserted through this needle using the Seldinger technique and the needle can be removed. Increasing dilators and short cuts on both sides can be used to widen the tracheostoma. The cannula can be placed and the guide wire is removed. After inflation of the cuff, a bronchoscopy via the cannula is performed to confirm its correct intratracheal position [4].

We report here a patient’s case, in which a severe bleeding occurred at the deep bifurcation between bronchus 9 and 10, most probably caused by the guide wire.

## Case

A 32-year-old male was admitted to the university hospital emergency department after being involved in a physical assault. On arrival at the hospital, he was initially able to walk but presented with anisocoria, became increasingly hypoxic and required intubation. After successful intubation, he became increasingly bradycardic and had to be resuscitated. After 3–4 min, a return of spontaneous circulation wasachieved. A computed tomography scan showed intracerebral hyperdensities.

Further assessment revealed various injuries from of the assault, including facial fractures, as well as hypoperfusion of some abdominal organs, probably as a result of resuscitation. He was then transferred to the surgical intensive care unit (ICU) with catecholamine support of 0.08 mcg/kg/min norepinephrine. Seven days after admission to the ICU, an orbitotomy with nerve decompression and treatment of the facial fractures was performed.

Ten days after admission to the ICU, PDT was scheduled due to inadequate awakening.

The continuous infusion of 500 IE/h unfractionated heparin since admission was stopped five hours before the procedure. The patient was not on antiplatelet therapy.

The coagulation laboratory results, except for platelets, were all within reference limits at the time of the procedure (Table 1).

**Table 1:** Laboratory results at the time the PDT was performed

parameter	value	reference
Quick, %	112	70–120
INR	1.0	depending on therapeutic goal
aPTT, sec	27	23–32
Thrombocytes, 10^9^/L	61	150–400

*aPTT: activated partial thromboplastin Time*,* INR: International Normalized Ratio*,* PDT: percutaneous dilatational tracheostomy.*

**Fig. 1:**
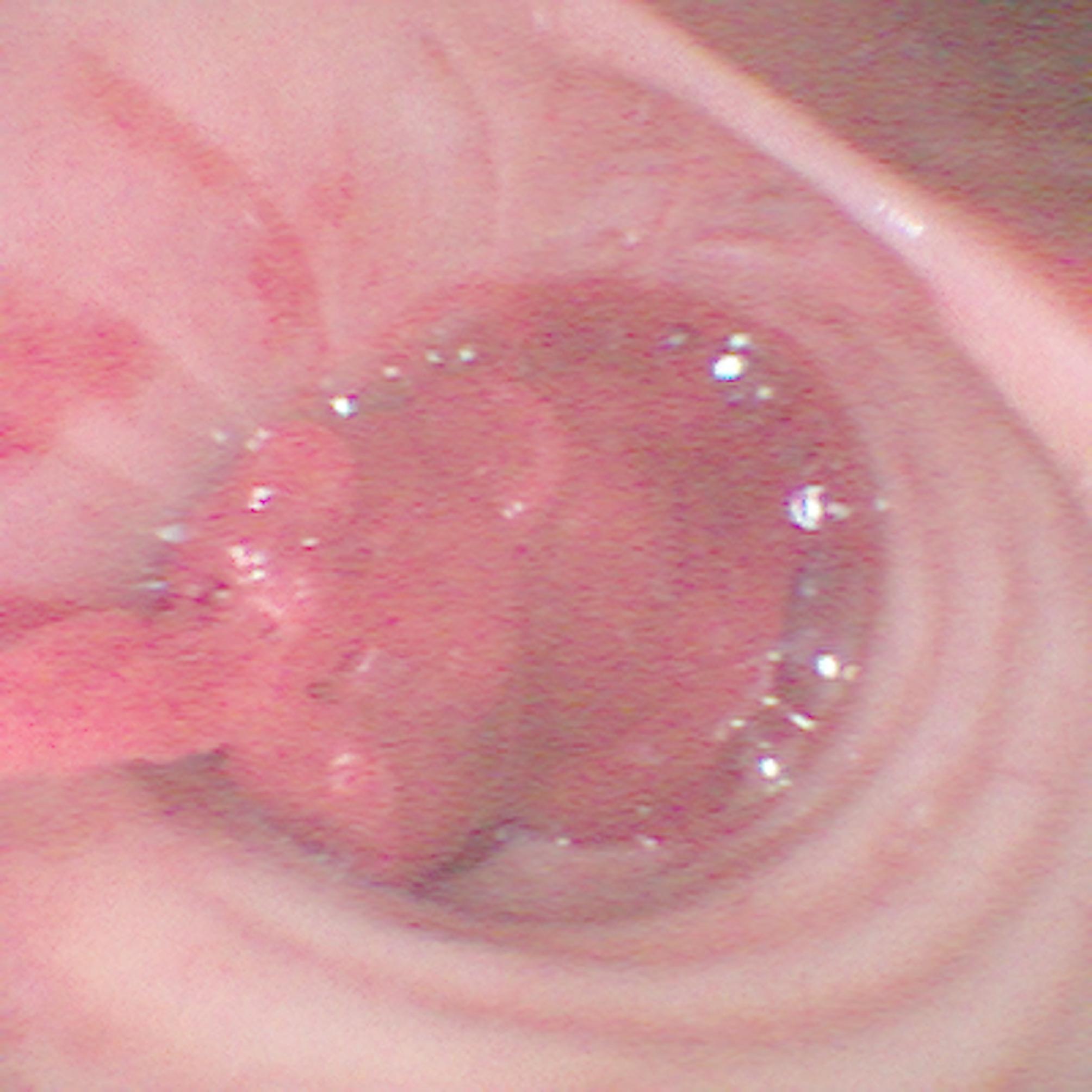
Blood clot in the right main bronchus

The procedure was performed at the bedside as described above using the Tracoe Experc Dilatation Set (Tracoe medical GmbH, Nieder-Olm, Germany) without any apparent complications. Immediately after PDT, the team found an active bleeding and a blood clot in the right main bronchus blocking the airway (Fig. 1). Epinephrine and tranexamic acid were applied through the working channel of the endoscope to the presumed location of the bleeding source. After incomplete retrieval of the clot, the bleeding was controlled, but the source of the bleeding could not yet be identified. Hypoxia never occurred.

**Fig. 2:**
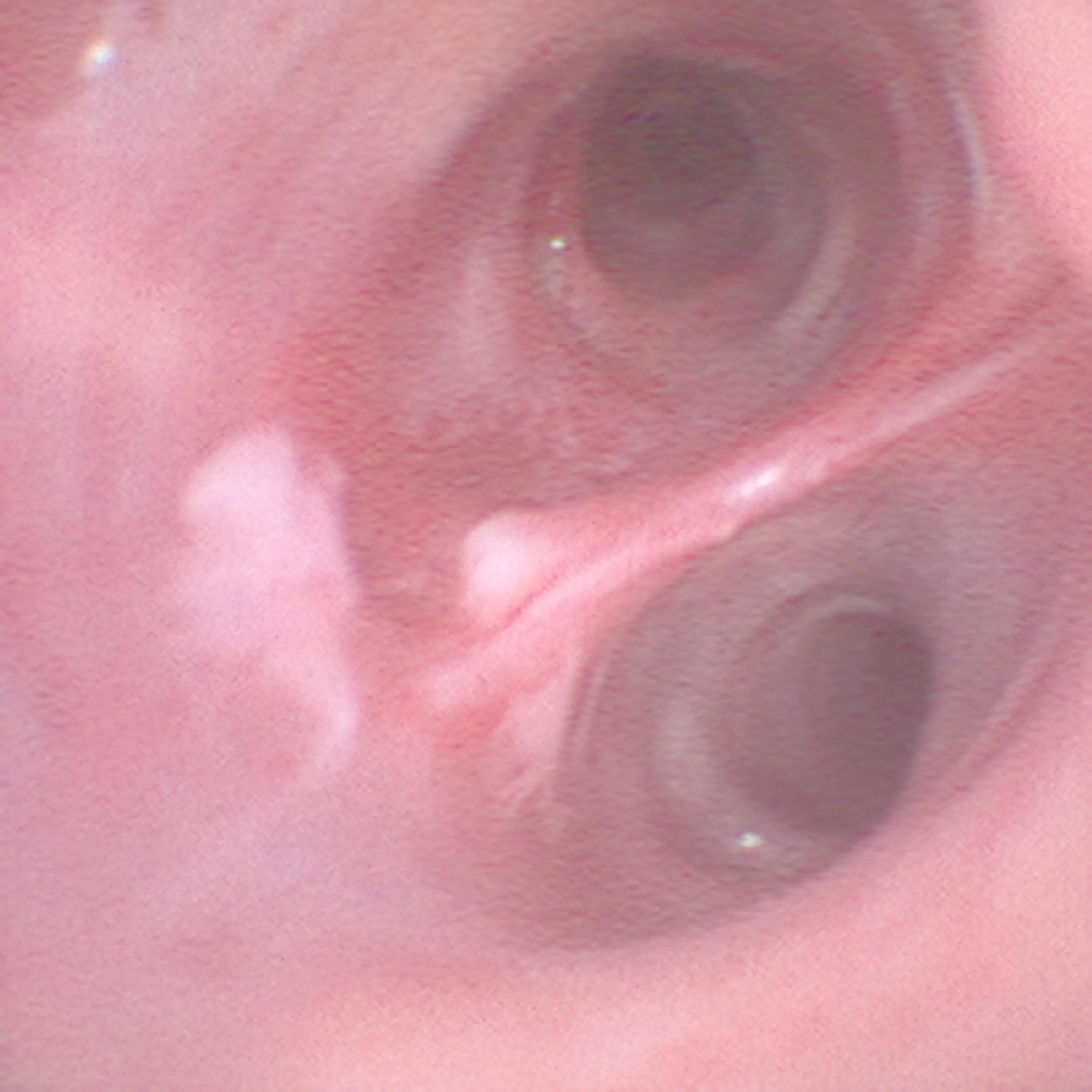
Mucosal lesion

After exposure of the right intermedial bronchus and observation of the lower lobe bronchus, a mucosal lesion was found at the bifurcation approximately between segments 9 and 10, which was suspected to be the source of bleeding (Fig. 2).

Adequate oxygenation was ensured, and after consultation with a pulmonary specialist, a conservative strategy was adopted.

**Fig. 3:**
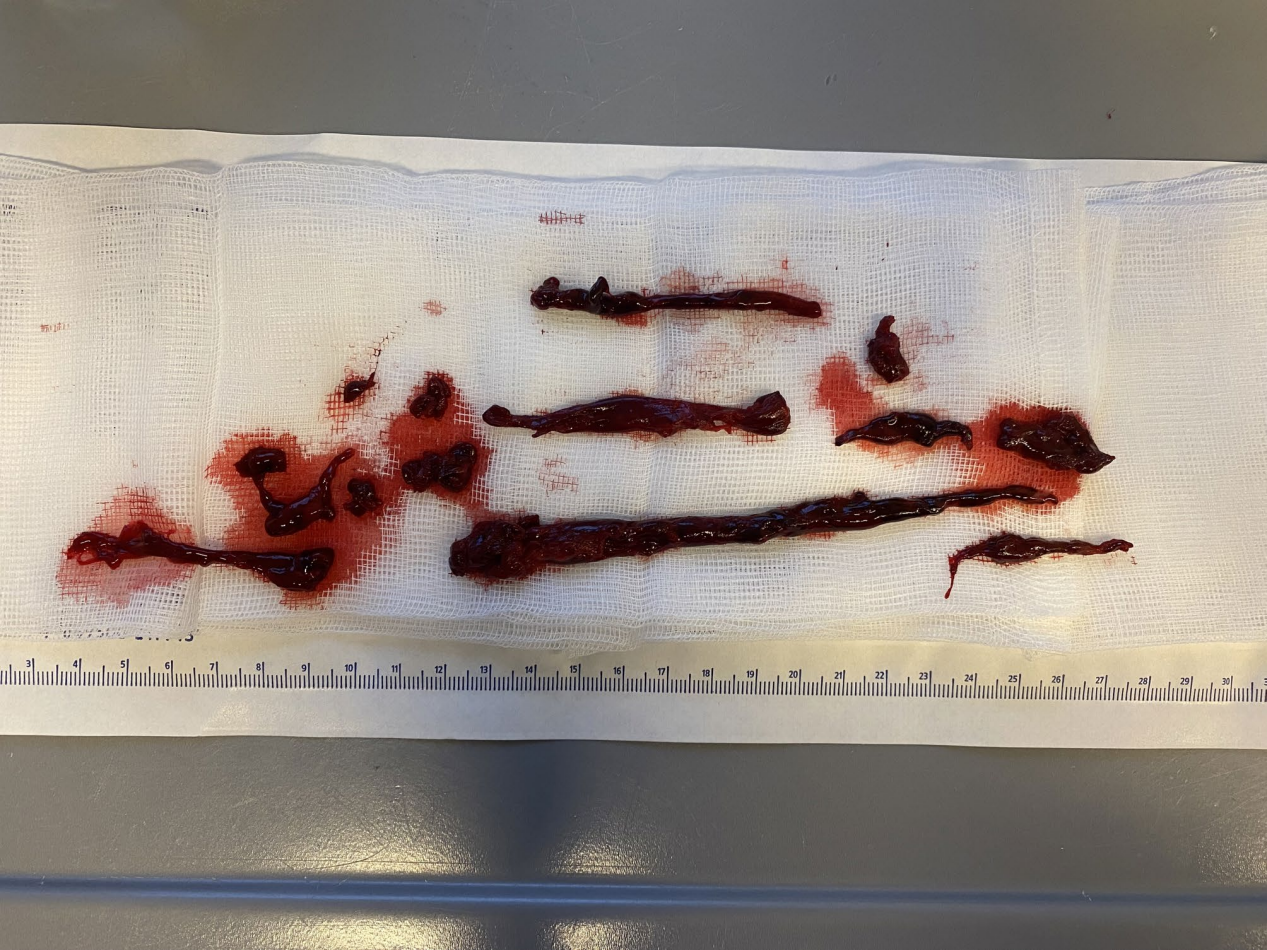
The individual pieces of blood clot removed from the bronchial system

Three days later, the remaining proportion of the clot could be, apart from a small clot, completely removed by bronchoscopy (Fig. 3). The patient was subsequently weaned from mechanical ventilation. 42 days after PDT, the cannula was removed and he had adequate lung function without supplemental oxygen.

### 3D printing

**Fig. 4:**
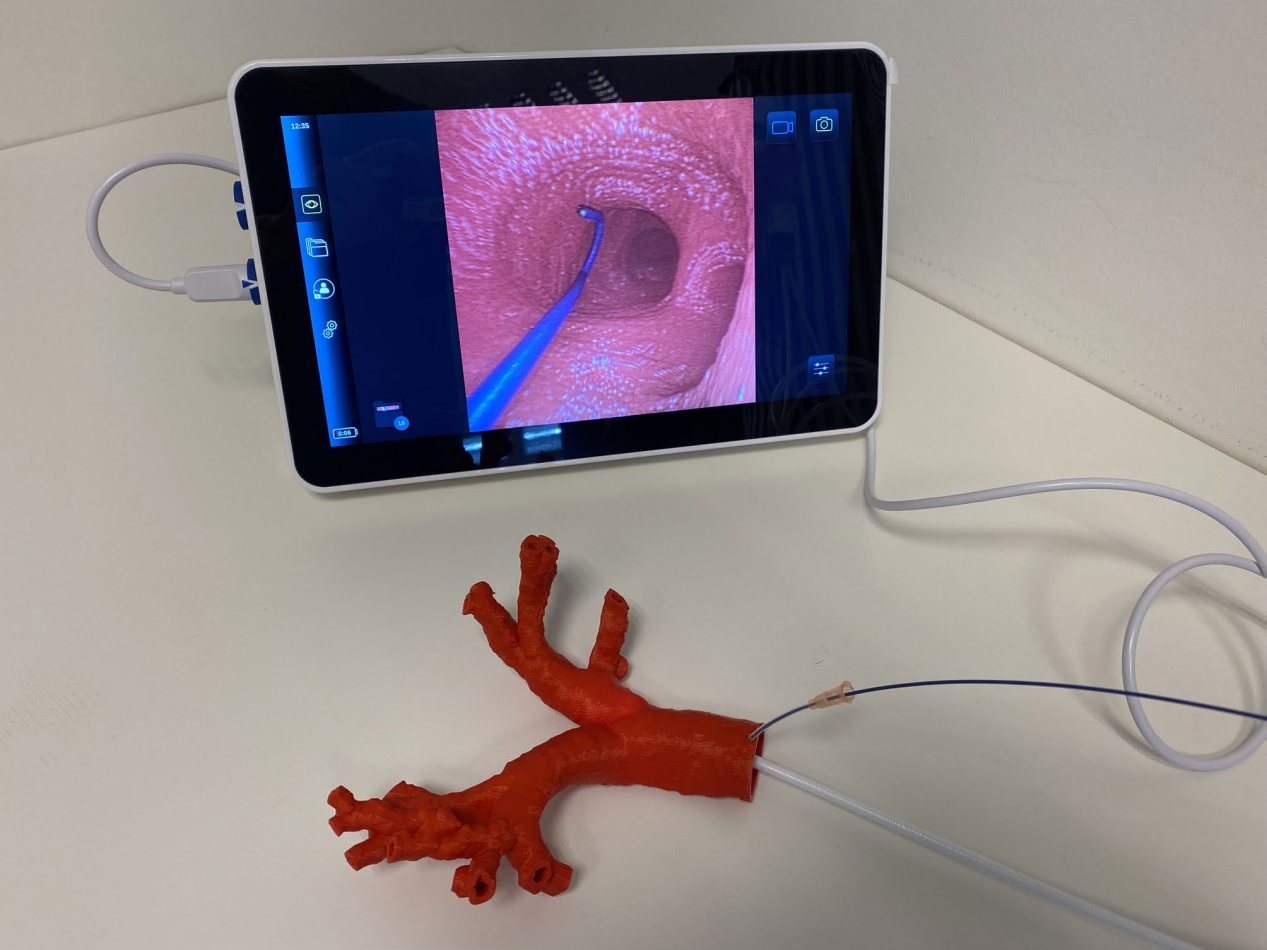
Simulation setup with lifelike three-dimensional model of the patient’s bronchial system

In the case described above, a patient-specific (1:1) 3D model of the patient’s bronchial system was printed (Fig. 4). Using a high-resolution tomography (CT)-scan of the patient’s chest (spatial resolution 1 mm), the bronchial system was segmented (Mimics Innovations suite, Materialise GmbH, Gilching, Germany) and a hollow model was generated (by adding 2 mm wall thickness). Subsequently, the model was printed on a high-resolution fused deposition-modeling printer (Ultimaker S5, Ultimaker B.V., Geldermalsen, Netherlands) with PLA polymer [5, 6]. There have been several applications in medicine like the 3D printing of prostheses and organs, applications in medical education and research, and planning of surgical procedures [7, 8]. It allows an anatomical depiction and can help to reconstruct challenging circumstances. We used the 3D-printed model to perform bronchoscopy (Fig. 4).

## Discussion

In this case report, we have described an unusual bleeding during a PDT that probably occurred during guide wire placement.

The main complications of PDT are bleeding, infection or hypoxia [9]. Due to the anatomical proximity, the jugular veins, the thyroid vessels, or even the brachiocephalic trunk can be injured [4]. The risk is between 7.4% and 29.4% and is increased with coagulopathies [10, 11]. Overall, the literature focuses on vascular injuries in anatomic proximity to the tracheostomy.

**Fig. 5:**
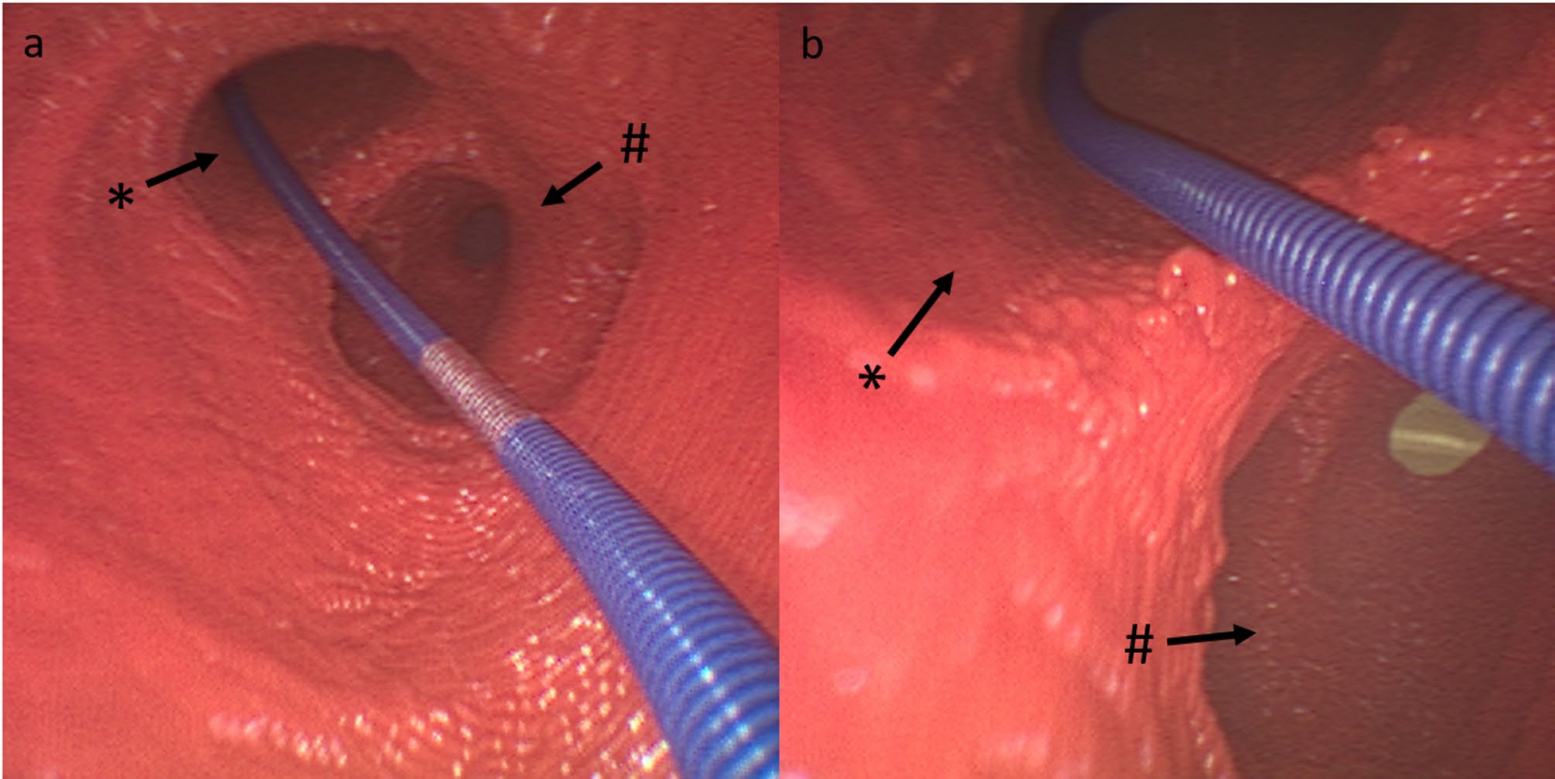
**(a)** Position of the guide wire at a bifurcation in the 3D model, **(b)** a close-up view. * Segment 9, # Segment 10

This case illustrates that bleeding complications can also occur more distally in the patient’s bronchial system which is both difficult to detect and to control. By clogging the bronchi and reducing the oxygenation surface, as little as 150–200 milliliters of blood can cause hypoxia [8]. In this case, the bleeding was most likely caused by an unperceived mucosal irritation by the guide wire at the bronchial bifurcation, approximately between segments 9 and 10, as shown in the 3D model simulation (Figs. 5). Anatomically, the V9 segmental vein can run in front of or behind the basal part of the right bronchus and flows into the inferior pulmonary vein. The segmental vein V10 is the terminal branch and begins between the segmental bronchi B9 and B10. It usually flows into the inferior pulmonary vein under the B10 segmental bronchus [12].

### Guide wire

This kink-resistant Nitinol guide wire (Ø 1.27 mm, Tracoe medical GmbH, Nieder-Olm, Germany) is bent in a J-shape to prevent direct perforation by its tip. However, this may mislead the medical staff into advancing the wire, causing the tip to disappear from view.

**Fig. 6:**
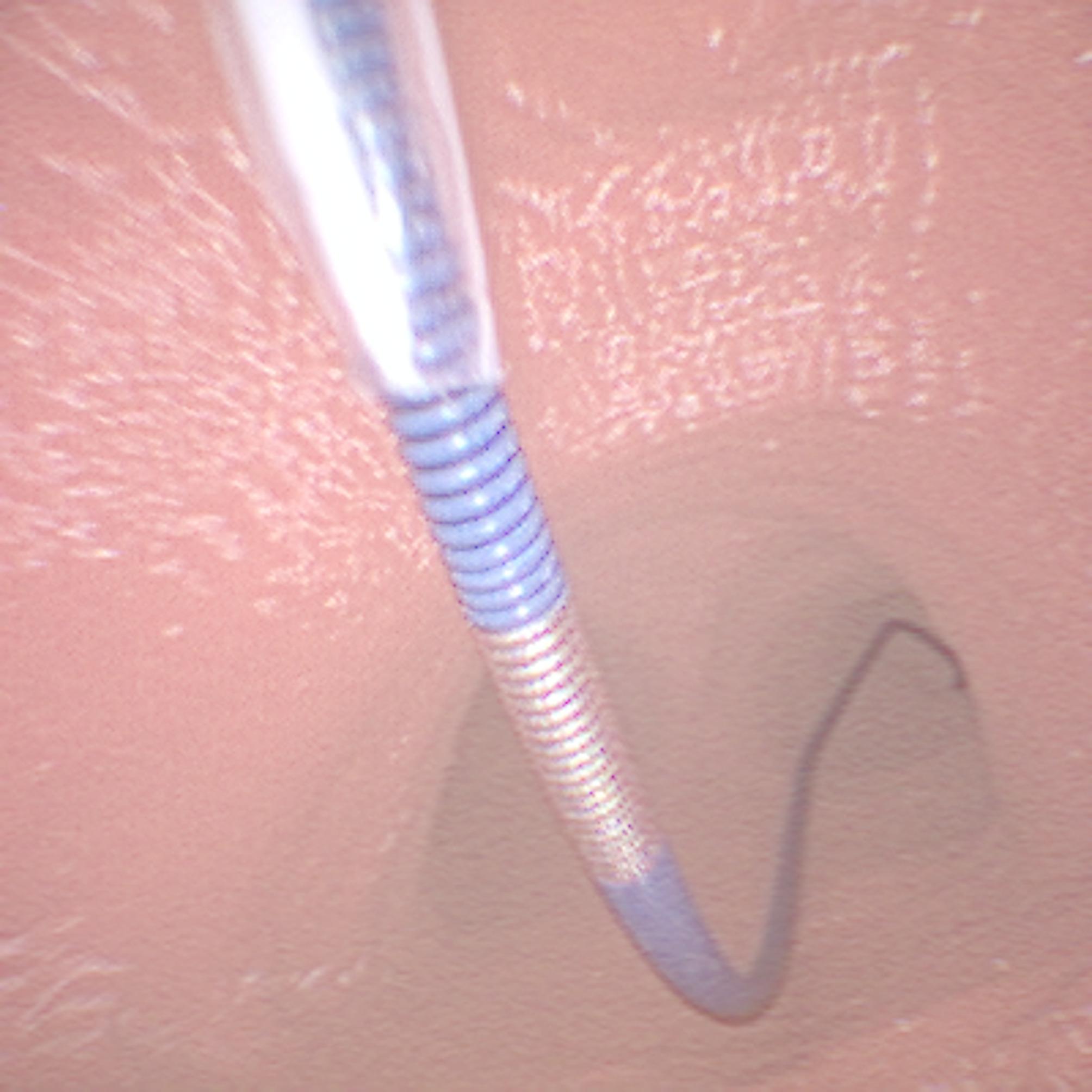
View of the guide wire through the bronchoscope after insertion into the 3D trachea model

Guide wires are typically marked approximately ten centimeters from the tip (Fig. 6).

The average length of the trachea is 11.8 cm [13]. Since the puncture for PDT is usually between the second and the third tracheal ring, ten centimeters should be a sufficient insertion depth.

The authors conclude that the contact between the tip and the carina alone is not critical, but that the tension of the wire, when it is threaded into a bronchus can lead to mucosal lesions (Fig. 5B). This highlights the following points that should be considered when performing PDT.

The marker can be used to confirm adequate intratracheal depth. In addition, the tip position can be confirmed bronchoscopically.

### Thrombocytopenia

Regarding thrombocytopenia, the health care professionals must weigh the risks and benefits of a PDT at this time. In a retrospective analysis, Pilarczyk et al. identified coagulopathies with low fibrinogen or thrombocytopenia as risk factors for bleeding after PDT [11].

Al Dawood et al. found that the incidence of bleeding after PDT remains low in patients with coagulopathy or thrombocytopenia (platelet count ≤ 60 × 10^9^ / L) [14]. In a retrospective, single-center cohort study, Kluge et al.. concluded that the procedure has a low complication rate when performed in patients with a platelet count of less than 50 × 10^9^ / L, which is lower than the level of the patient in this case report [15].

The guideline of the German Medical Association recommends the administration of platelets in a transbronchial biopsy, if the platelet count drops below 50 × 10^9^ / L [16]. The lesion in this case is comparable to a transbronchial biopsy. Given the patient’s normal coagulation lab results, the recommendations, and the clinic’s standard operating procedure, the medical team concluded that it was safe to proceed with PDT. Accordingly, thrombocytopenia is not a contraindication to PDT, but it is most likely to have increased the bleeding in this case.

### 3D printing

A 3D model was created from a CT scan of the patient which allowed us to demonstrate the etiology of the bleeding and to link the initially unknown source of the bleeding to specific anatomical structures to prevent such a complication in the future.

Thus, 3D printing can be a valuable tool for demonstrations and error management.

## Conclusion

During PDT, unperceived bleeding complications may occur more distally in the bronchial system. These injuries to the bronchial system are rare but can be potentially life-threatening. Therefore, the guide wire should be inserted not further than necessary during the procedure. Ten centimeters is a valuable depth that should be observed with caution. In addition, 3D printing can help to better understand the course of events and provide opportunities for improvement in future cases.

## Data Availability

No datasets were generated or analysed during the current study.

## Abbreviations

CT: Computer tomography
ICU: Intensive care unit
PDT: Percutaneous dilatational tracheostomy
